# The Number of Negative Lymph Nodes is Positively Associated with Survival in Esophageal Squamous Cell Carcinoma Patients in China

**DOI:** 10.1515/med-2020-0023

**Published:** 2020-03-08

**Authors:** Lan Yu, Xiao-Tao Zhang, Shang-Hui Guan, Yu-Feng Cheng, Lin-Xi Li

**Affiliations:** 1 Department of Radiotherapy, Qilu Hospital of Shandong University, No 107, Wenhua West Road, Lixia District, Jinan 250012, Shandong, China; 2 School of Medicine, Shandong University, Jinan 250012, China; 3 Cancer Prevention Center, Qingdao Central (Cancer) Hospital, The Second Affiliated Hospital of University Qingdao, Qingdao 266042, China; 4 Queen Mary School, Medical college of Nanchang University, Nanchang 330088, China

**Keywords:** Esophageal squamous cell carcinoma, Lymph node, Esophagectomy, Metastasis, Prognosis

## Abstract

**Background:**

There is a controversial relationship between the negative lymph nodes (NLNs) and survival in patients with esophageal squamous cell carcinoma (ESCC). This study investigates the implications of total number of NLNs on thoracic ESCC patient prognosis.

**Methods:**

579 thoracic ESCC patients were categorized into four groups (0-9, 10-14, 15-19 and ≥20 NLNs). Univariate analysis was done by the log-rank tests while multivariate analysis was undertaken using Cox regression models. Survival analysis was determined employing the Kaplan-Meier method.

**Results:**

When the numbers of NLNs were 9 or less, 10 to 14, 15 to 19 and 20 or more, patients of 3-year survival rates were 21.7%, 40.0%, 61.2% and 77.5%, respectively (*P*<0.001). In the node-negative and node-positive subgroups, 3-year survival rates were 34.9% and 14.3%, 50.9% and 19.3%, 65.6% and 51.8%, 81.4% and 68.9% respectively (*P*<0.001). Gender, tumor length, tumor differentiation, T and N stage as well as the total NLNs were found to be significantly linked to survival rates. Multivariate analysis showed tumor length, T stage, N stage and total NLNs were independent prognostic factors for ESCC patients.

**Conclusion:**

NLNs numbers is a significant independent prognostic indicator for thoracic ESCC patients’ survival after curative esophagectomy.

## 1 Introduction

Esophageal carcinoma (EC) is a frequently encountered cancer and a major contributor of deaths attributed to cancer worldwide [1]. This condition is found at the highest rates in Eastern Asia as well as in Eastern and Southern Africa [1]. In 2015, China reported an approximate 477,900 newly diagnosed EC patients and 375,000 EC-related mortalities [2]. The 2 main pathological types of esophageal cancer are adenocarcinoma and squamous cell carcinoma (SCC). Upwards of 90% of patients with EC have the SCC subtype, especially in populations such as China where EC is rampant [3]. Despite advancements in multidisciplinary treatment in recent years, complete resection is still the only treatment that can cure EC. Previous literatures have suggested that the total metastatic lymph nodes (LNs) represents an independent prognostic factor in determining EC patients’ survival rates [4, 5]. Although the 7th American Joint Committee on Cancer (AJCC) staging system recommends that a total dissection of at least 15 LNs are necessary to accurately stage patients undergoing esophagectomy without induction chemoradiation, there are controversies about the optimal extent of LN dissection during radical lymphadenectomy [6, 7].

In recent years，the number of NLNs has been confirmed to be an important factor related to patient survival in many cancers, proving to be more accurate than the number of positive LNs in predicting patients’ prognosis [8, 9, 10]. Several papers have scrutinized the impact of NLNs on patient survival for EC [11, 12, 13]. Greenstein et al. [12] found that an increasing NLNs count was related to with long-term patient survival in those with lymph node-negative esophageal adenocarcinoma but not in those with the squamous cell subtype. Another study demonstrated that NLNs count was an advantageous prognostic indicator in lymph node-positive EC patients but not in those who were lymph node-negative [13]. Wu et al. [14] draw the conclusion that the number of NLNs was an independent prognostic factor for node-negative EC through a retrospective review of 429 EC patients who underwent modern two-field lymphadenectomy. These results are controversial and need to be further investigated. In the present investigation, we assessed the relationship between the number of NLNs and prognosis of patients with ESCC after curative esophagectomy.

## 2 Methods

### 2.1 Study population

579 patients diagnosed with thoracic ESCC who underwent curative esophagectomy at the Department of thoracic surgery, Qilu hospital, Shandong University, China, between January 2009 and December 2012 were involved in this retrospective analysis. Patient ages ranging between 40 to 70 years; a diagnosis of thoracic ESCC (confirmed via histopathology); had not received preoperative chemotherapy and/or radiotherapy; no second primary tumor history; comprehensive preoperative examination including upper gastrointestinal imaging, chest and upper abdomen enhanced computed tomography, neck color doppler ultrasonography and gastroscope; complete resection(R0); complete clinical data and at least 3 years of follow-up or death; survival time more than three months were all inclusion factors for this study. The exclusion criteria were as follows: patients with non-cancer related death; no R0 resection; cervical esophageal cancer or those lost to follow-up. This study was approved by the Institutional Review Board of the Qilu hospital, Shandong University. All patients obtained informed consent and signed the informed consent form.

There were 579 cases including 486 male and 93 female participants. All patients were aged between 40 to 70 years with a median age of 61 years. There were 51 cases with upper thoracic tumors, 227 cases with middle thoracic tumors and 301 cases with lower thoracic tumors. Based on histological examination, 92 cases were determined to be well differentiated, 256 were moderately differentiated, while 231 were poorly differentiated. Based on the 7th edition of AJCC classification, there were 79 cases (13.6%) for stage I, 307 cases (53.0%) for stage II and 193 cases (33.3%) for stage III.

### 2.2 Surgical procedures

All 579 patients underwent trans-thoracic esophagectomy and lymphadenectomy, including two-field (mediastinal and abdominal) LN dissection or three-field (mediastinal, abdominal and cervical) LN dissection. The NLN is the LNs retrieved during radical lymphadenectomy which were not found to have tumor cell metastasis in pathological examination. The number of NLNs is the quantity of LNs retrieved during radical lymphadenectomy minus the count of metastatic LNs.

### 2.3 Follow-up

Follow-up was performed by means of out-patient review, hospital review, phone calls and letters after surgery. Patients were followed-up every quarter for the initial two years, biannually from years three to five and annually thereafter. All patients received a comprehensive medical examination including routine blood investigations, liver and kidney function, upper gastrointestinal imaging, chest and upper abdomen enhanced computed tomography, neck color Doppler ultrasonography, gastroscope, brain magnetic resonance and bone scintigraphy imaging when they were followed up. In this study, all subjects were monitored for at least three years post-surgery or until death. Patients who failed to meet this requirement were labeled as lost to follow-up and were excluded from the study. In the end, 579 patients met the criteria for inclusion in this study. Data collection concluded on December 2015.

### 2.4 Statistical analysis

Log-rank tests were used to carry out univariate analysis while Cox regression models were employed for multivariate analysis. Patient survival was defined as the surgery date to the last point of follow-up or death, and was determined using the Kaplan-Meier method. A *P*-value<0.05 was taken to indicate statistical significance. All calculations were carried out employing the SPSS program version 23 (SPSS, IBM, USA).

Univariate analysis variables comprised of age (≤60 years and >60 years), gender (male or female), tumor length (<4 cm and ≥4 cm), location of the tumor (upper, middle and lower), tumor differentiation (poorly, moderately and well differentiated), T stage (T1, T2, T3 and T4), N stage (N0, N1, N2, and N3) as well as the number of NLNs (0-9, 10-14, 15-19 and ≥20 NLNs). Multivariate analysis involved tumor length, T stage, N stage and the number of NLNs.

## 3 Results

### 3.1 Long-term surgical outcomes

All 579 patients were followed up for a median of 36 months (total range between 3 to 80 months). Overall, patients had an average survival time of 33.9 months and 50.8% (294/579) of them had a 3-year survival rate. The 3-year survival rates of patients with stage I, stage II and stage III were 86.1% (68/79), 53.7% (165/307) and 31.6% (61/193) respectively (χ2 = 71.864, *P*<0.001).

### 3.2 Total number of LNs dissected

The total quantity of LNs dissected for all patients was 8015 (range, 0-53; median 12; mean 12.7±7.8) and the total number of NLNs for the entire cohort was 7366 (range, 1-53; median 13; mean 13.8±8.2). There were 344 node-negative patients whose total number of LNs dissection was 4627 (range, 1-41; median 12; mean 13.5±8.0) and 235 node-positive patients whose total number of LNs dissection was 3388(range, 1-53; median 14; mean 14.2±8.3).

### 3.3 The effect of total LNs on node-negative patient survival

**Figure 1:**
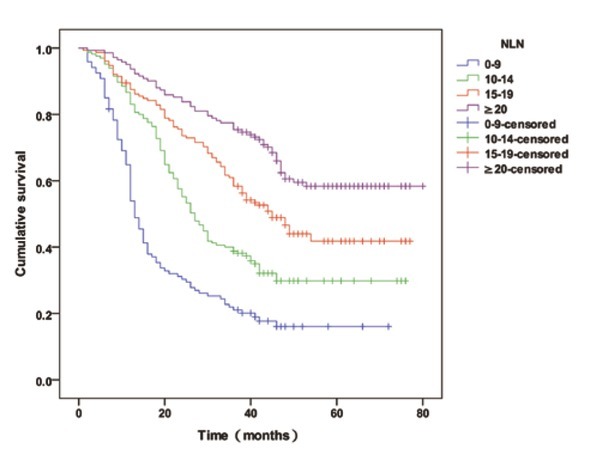
The overall survival curve for all patients with 9 or less, 10 to 14, 15 to 19 and 20 or more NLNs

All participants were grouped into 4 categories as follows, based on the number of NLNs: 0-9 NLNs (120 cases), 10-14 NLNs (165 cases), 15-19 NLNs (152 cases) and ≥20 NLNs (142 cases). When the numbers of NLNs were 9 or less, 10 to 14, 15 to 19 and 20 or more, the median survival time was 13 months, 27 months, 39 months and 47 months (*P*<0.001) respectively, and the 3-year patient survival rates were 21.7% (26/120), 40.0% (66/165), 61.2% (92/152) and 77.5% (110/142) respectively（*P*<0.001), as shown in Table 1 and Fig 1.

**Table 1:** The survival characteristics of the node-negative and node-positive subgroups.

Number of NLNs	All patients(n=579)			node-negative patients(n=344)			node-positive patients(n=235)		
	No	MST(m)	3-year survival (%)	No	MST(m)	3-year survival (%)	No	MST(m) 3-year survival (%)	
0-9	120	13	21.7% (26/120)	43	18	34.9% (15/43)	77	12	14.3% (11/77)
10-14	165	27	40.0% (66/165)	108	36	50.9% (55/108)	57	22	19.3% (11/57)
15-19	152	39	61.2% (92/152)	96	42.5	65.6% (63/96)	56	36	51.8% (29/56)
≥20	142	47	77.5% (110/142)	97	48	89.0%(79/97)	45	45	68.9% (31/45)

### 3.4 The effect of the number of NLNs on node-negative patient survival

**Figure 2:**
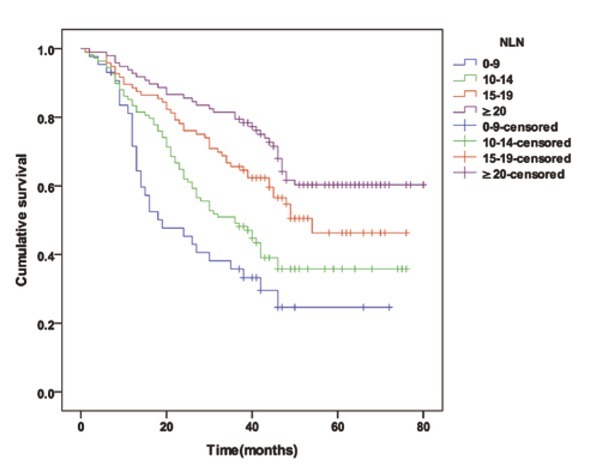
The overall survival curve for node-negative patients with 9 or less, 10 to 14, 15 to 19 and 20 or more NLNs

There were 344 node-negative patients who were categorized into 4 cohorts as follows, based on the number of NLNs: 0-9 NLNs (43 cases), 10-14 NLNs (108 cases), 15-19 NLNs (96 cases), ≥20 NLNs (97 cases). When the numbers of NLNs were 9 or less, 10 to 14, 15 to 19 and 20 or more, the median survival time (MST) was 18 months, 36 months, 42.5 months and 48 months (P<0.001) and the 3-year survival rates of patients were 34.9% (15/43), 50.9% (55/108), 65.6%（63/96）and 89.0%（79/97）respectively (*P*<0.001), as shown in Table 1 and Fig 2.

### 3.5 The effect of the number of NLNs on node-positive patient survival

**Figure 3:**
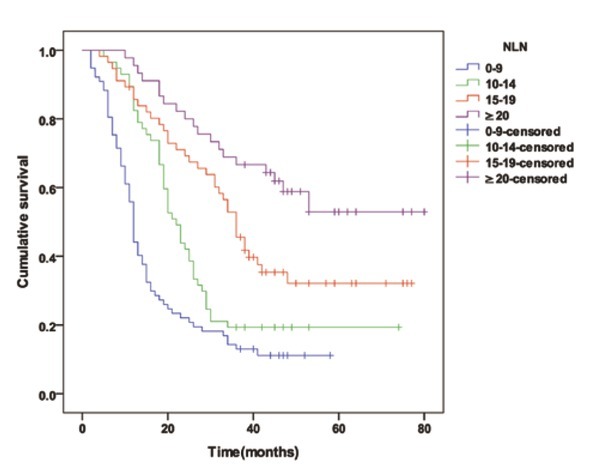
The overall survival curve for node-positive patients with 9 or less, 10 to 14, 15 to 19 and 20 or more NLNs

There were 235 node-positive patients who were categorized into 4 cohorts as follows, based on the number of NLNs: 0-9 NLNs (77 cases), 10-14 NLNs (57 cases), 15-19 NLNs (56 cases), ≥20 NLNs (45 cases). When the numbers of NLNs were 9 or less, 10 to 14, 15 to 19 and 20 or more, the MST was 12 months, 22 months, 36 months and 45 months (*P*<0.001) and the 3-year survival rates of patients were 14.3% (11/77), 19.3% (11/57), 51.8% (29/56) and 68.9% (31/45) respectively (*P*<0.001), as shown in Table 1 and Fig 3.

### 3.6 Univariate and multivariate survival analysis

Univariate analysis indicated that significant factors that influences the 3-year overall survival (OS) after esophagectomy were gender (*P*=0.023), tumor length (*P*=0.000), tumor differentiation (*P*=0.011), T stage (*P*=0.000), N stage (*P*=0.000) as well as the number of NLNs (*P*=0.000) (Table 2). Further analysis using multivariate methods indicated that significant factors affecting the 3-year OS after esophagectomy were tumor length (*P*=0.042), T stage (*P*=0.000), N stage (*P*=0.000) and the number of NLNs (*P*=0.000) (Table 2).

**Table 2:** Univariate and multivariate analysis of prognostic factors for overall survival.

Characteristics	N	3-year survival (%)	Univariate analysis		Multivariate analysis	
			χ2	P	HR (95%CI)	P
Sex			5.163	0.023		
Male	486	49.0%				
Female	93	60.2%				
Age			0.479	0.489		
<60	260	52.3%				
≥60	319	49.5%				
Tumor location			0.808	0.668		
Upper	51	47.1%				
Middle	227	52.4%				
Lower	301	50.2%				
Tumor length(cm)			19.466	0.000		0.042
≤4	365	57.5%			Reference	
>4	214	39.3%			1.260(1.009-1.575)	
Tumor differatiation			9.019	0.011		
Poorly differatiated	231	48.1%				
Moderately differatiated	256	48.0%				
Well differatiated	92	65.2%				
T stage			49.654	0.000		0.000
T1	54	94.4%			Reference	
T2	167	59.9%			2.344(1.268-4.332)	
T3	353	40.2%			3.363(1.848-6.119)	
T4	5	20.0%			4.565(1.418-14.700)	
N stage			62.587	0.000		0.000
N0	344	61.9%			Reference	
N1	141	39,7%			1.550(1.205-1.994)	
N2	71	26.8%			2.021(1.467-2.785)	
N3	23	26.1%			2.135(1.317-3.461)	
Number of NLNs			120.475	0.000		0.000
0-9	120	21.7%			Reference	
10-14	165	40.0%			0.500(0.378-0.662)	
15-19	152	61.2%			0.314(0.232-0.426)	
≥20	142	77.5%			0.199(0.141-0.280)	

NLN, negative lymph nodes; HR, hazard ratio; CI, confidence interval.

## 4 Discussion

The LN metastasis is a significant prognostic indicator for ESCC [15]. However, the extent of dissected LNs may influence pathological node staging and subsequently affect the prognosis of ESCC patients. Indeed, current literature demonstrates that the amount of LNs dissected is a significant prognostic factor for ESCC patients after curative esophagectomy [16, 17].

Several studies confirmed the number of NLNs was more accurate than the number of positive LNs in predicting patients outcomes. Nevertheless, there is still controversy between the number of NLNs and survival in EC patients. One of several studies investigating the number of NLNs and its effect on EC patient survival, Zhu et al. [18] studied 332 patients with thoracic ESCC who had at least 15 or more LNs dissected via three-field lymphadenectomy(3FLND) retrospectively and revealed that patients that possessed higher counts of NLNs were more likely to survive longer in contrast to those who had less NLNs. This was found to be true in both node-positive and -negative patients. Therefore, this investigation concluded that NLN number may serve to be able to independently prognosticate ESCC patients who received 3FLND. Another study using data of 972 patients with node-negative EC extracted from the SEER (Surveillance, Epidemiology and End Results) database investigated the NLNs number on their postoperative survival. This study revealed that an elevated count of NLNs was directly proportional with increased patient survival in those with EC adenocarcinoma subtype, but not in those with squamous cell subtype [12]. Baba et al. [11] investigated 252 patients with surgically resected ESCC from a single center and demonstrated that the number of NLNs carried positive prognostic value in patients with positive LNs but not with those with negative LNs.

In this retrospective analysis, we evaluated the impact of NLNs number on the survival of a total of 579 thoracic ESCC patients from a single center who all received curative esophagectomy. Ma et al. [13] demonstrated that ESCC patients with more than 20 NLNs have better survival rates in contrast to those with less than or equal to 20 NLNs, through retrospective analysis of 381 patients who had underwent surgical resection. Zhang et al. [19] retrospectively analyzed 99 patients who were identified with middle thoracic ESCC after esophagectomy and found obvious differences of OS between the following groups: patients with more than and less than 10 NLNs and patients with＞15 and ⩽15 NLNs. Based on the above research evidences, we categorizes the NLNs into 4 groups (0-9, 10-14, 15-19 and ≥20 NLNs) to further explore the relationship between the number of NLNs and prognosis of patients with ESCC after curative esophagectomy. When the number of NLNs were 9 or less, 10 to 14, 15 to 19 and 20 or more, the 3-year survival rate of patients were 21.7%, 40.0%, 61.2% and 77.5% respectively (*P*=0.000). In the node-negative subgroup and the node-positive subgroup, 3-year survival rate were 34.9% and 14.3%, 50.9% and 19.3%, 65.6% and 51.8%, 81.4% and 68.9% respectively (*P*=0.000). We found that thoracic ESCC patients with higher numbers of NLNs were more likely to have better survival rates in contrast to those with less NLNs, irrespective of their node-positive or -negative status. This concurs with the results of another retrospective study on 381 ESCC patients who also received tumor resections that demonstrated that the total NLNs were strongly linked with overall patient survival time. In this study, 45.4% of patients with more than 20 NLNs passed the 5-year survival rate mark, while only 26.4% of 239 patients with less than or equal to 20 NLNs (*P*=0.001) had a 5-year survival rate [13]. Zhang et al. (19) found the total NLNs count was more significant than any other parameters and the overall survival time and determined that those with less than 5 NLNs had markedly different survival times in comparison to those who had more than 5 NLNs. This finding was also true at threshold numbers of 10 and 15 NLNs. Moreover, in this study, univariate analysis indicated that gender, tumor length, tumor differentiation, T and N stage as well as the total of NLNs served as independent prognostic factors. Only tumor length, T and N stage as well as total NLNs appeared as independent prognostic factors after subsequent multivariate analysis. Similarly, multivariate analyses by Ma et al. [13] on ESCC patients also supported TNM factors as well as the total NLNs dissected to be independent prognostic factors in determining patients’ fates.

The results from our study was similar to those of Zhu et al, but was different from Baba et al, Ma et al, and Greenstein et al. The first possible potential reason is the inconsistency of enrolled population and the inclusion criteria. The second possible reason is the differences of surgical approach and extent of lymph node dissection in researches of different years, and the third possible potential reason is the differences of grouping criteria for NLNs.

Our study did not identify the potential mechanism underlying survival and total number of NLNs. Several theories have been put forward by some researchers. The first theory is stage migration which indicates that dissecting more LNs may uncover more node metastases, which can provide more accurate information on pathological node staging and reduce the possibility of inaccurate staging [20]. One population-based propensity score-adjusted study demonstrated that more retrieved regional LNs in pancreatic cancer decreased the rate of stage migration and improved the oncological outcome in node-negative and -positive cancer [21]. Woo et al. [22] found that more accurate staging was achieved with more LN resection, while inadequate LN dissection is implicated in under staging of patients. Researchers have previously demonstrated that immunohistochemical analyses have shown that a high rate of LN-negative patients have nodal micrometastases which were missed during routine pathology examinations [23]. Thus, increased number of removed NLNs could eliminate some potential remnant lesions and reduce the potential of micrometastases and subsequent tumor recurrence and metastasis. This also explains why patients with more NLNs had better survival in our study. Our study also demonstrates less NLNs is related to poorer survival, supporting the hypothesis of stage migration. The second explanation is that an increased number of NLNs reflects a stronger immune reaction to the tumor. He et al. [24] found a high number of NLNs was an indicator of greater lymphocyte infiltration, not only in the invasive central but also in the margin region of tumor microenvironment. The favorable prognosis brought about by higher numbers of NLNs could reflect the immune response of host lymph node cells to tumor cells, which is related to LN count. In addition, lymph nodes response to primary tumors has been proven to be linked to longer patient survival in the case of those with colorectal cancer [25]. Zhang et al. [26] discovered that the LN status at station 108 may indicated the prognosis of ESCC patients, and NLN may reflected the reaction of the immune system to tumor metastasis in these patients. The third hypothesis is that a greater number of retrieved LNs could be a representative of the proficiency and quality of surgical treatment and subsequent pathological examination [27].

There are three limitations in our study. Firstly, patients in our study came from the same institution, thus our findings may not be applicable to the general population. The potential reasons may be that there are many significant geographical differences for EC [28]. In addition, there may have regional differences in surgical approach and the extent of LN dissection. Second, the study does not provide information on the effect of adjuvant chemotherapy and radiotherapy on patients, both of which may affect patient prognosis. Thirdly, our study was not able to adequately explore the mechanistic relationship between patient survival and number of NLNs, and was also not able to define the optimal number of NLNs for ESCC patients.

Nevertheless, this investigation supports NLNs number as a factor able to independently prognosticate patients with patients with thoracic ESCC. Therefore, to improve survival rates, we suggest that more LNs should be retrieved to increase the number of NLNs under the premise of controlling surgical complications. Future studies are necessary to identify the potential mechanism underlying the link between patient survival and the number of NLNs as well as to explore the optimal NLN dissection number for ESCC.
